# Farewell message from the Editor-in-chief

**DOI:** 10.20945/2359-3997000000573

**Published:** 2022-11-28

**Authors:** Marcello D. Bronstein

**Affiliations:** 1 Hospital das Clínicas, Faculdade de Medicina da Universidade de São Paulo Serviço de Endocrinologia e Metabologia Chefe da Unidade de Neuroendocrinologia São Paulo SP Brasil Editor-Chefe da Archives of Endocrinology and Metabolism, Chefe da Unidade de Neuroendocrinologia, Serviço de Endocrinologia e Metabologia, Hospital das Clínicas, Faculdade de Medicina da Universidade de São Paulo (HCFMUSP), São Paulo, SP, Brasil

**DEAR COLLEAGUES,**

After 8 years and feeling accomplished, I am leaving the role of Editor-in-chief of our beloved *Archives* *of* *Endocrinology* *and* *Metabolism* – AE&M. Invited by the former president of SBEM, Dr. Nina Rosa Musolino, I accepted this mission proposing some changes, starting with altering the written language to English, seeking greater entrance in international endocrinology. In this way, the *Arquivos Brasileiros de Endocrinologia e Metabologia* became the *Archives of Endocrinology and Metabolism*.

At the time I was criticized. But I argued that the Journal would continue to be the official publication of SBEM, just like other journals that represent the scientific community of their countries and are also published in English, which, by the way, it became the universal scientific language (1). Over time, this decision proved to be accurate and appropriate, as our impact factor increased from 0.6 to 2,032. AE&M grew significantly. Something else that also contributed to this increase was the encouragement for the submission of review articles and the reduction of case reports.

The migration from paper to the online version, an increasingly sustainable practice, was also positive.

With the online publication and changing to English as the official language, we started to receive from Brazil and abroad, a significantly greater number of article submissions. Total of 4,080; 646 being accepted!

I am sure that our Journal will continue to grow with the new Editor-in-chief, Dr. Beatriz D'Agord Schaan, considering her competence and commitment to Science and, from now on, I am available to help in any way necessary.

Finally, I believe that we would not have achieved these goals without the collaboration of the editorial assistants, reviewers, my co-editors and, of course, SBEM. Not to mention you, dear colleague, who honored us by reading and submitting your articles.

Thank you very much. I wish that AE&M continue to reflect more and more the advances in Endocrinology and Metabology worldwide!
